# Impact of RAS mutation status on early progression in patients with initially unresectable colorectal liver metastases undergoing conversion therapy: a retrospective analysis

**DOI:** 10.3389/fsurg.2026.1853947

**Published:** 2026-07-01

**Authors:** Rong Yang, Weili Zhang, Jin Lan, Weihao Li, Zhigang Hong, Jun Chi, Jianhong Peng, Cong Li, Xiaojun Wu

**Affiliations:** 1 State Key Laboratory of Oncology in South China, Collaborative Innovation Center for Cancer Medicine, Sun Yat-sen University Cancer Center, Guangzhou, China; 2 Department of Intensive Care Unit, Sun Yat-sen University Cancer Center, Guangzhou, China; 3 Department of Colorectal Surgery, Sun Yat-sen University Cancer Center, Guangzhou, China; 4 Department of Endoscopy, Sun Yat-sen University Cancer Center, Guangzhou, China

**Keywords:** colorectal cancer, conversion therapy, early progression, liver metastases, RAS mutation

## Abstract

**Background:**

Colorectal cancer liver metastases (CRLM) frequently recur or progress after systemic and local treatment, particularly in patients with initially unresectable disease. RAS mutations are associated with poor outcomes in CRLM, but their relationship with early progression or recurrence after conversion therapy remains unclear. This study evaluated the prognostic impact of RAS mutation status in patients with initially unresectable CRLM (IU-CRLM) undergoing conversion therapy.

**Methods:**

This retrospective cohort study included 194 patients with initially unresectable, pMMR CRLM treated at Sun Yat-sen University Cancer Center between December 2012 and January 2020. Early progression/recurrence was defined as disease progression after failed conversion, recurrence after successful conversion, or death within six months. Event-free survival (EFS) and overall survival (OS) were analyzed according to RAS status.

**Results:**

Of 194 patients, 87 (44.8%) achieved successful conversion and 51 (26.3%) harbored RAS mutations. RAS-mutant patients had a higher early progression/recurrence rate than RAS wild-type patients (41.2% vs. 19.6%, *P* = 0.004). In patients with failed conversion, RAS mutation was associated with a higher early progression rate (36.7% vs. 13.0%, *P* = 0.012), whereas the early recurrence rate did not differ significantly by RAS status among successfully converted patients (47.6% vs. 27.3%, *P* = 0.142). In the clinically adjusted logistic model, RAS mutation remained independently associated with early progression/recurrence (OR: 3.546, 95% CI: 1.290–9.752, *P* = 0.014).

**Conclusions:**

RAS mutation is associated with a higher risk of early progression/recurrence in patients with IU-CRLM undergoing conversion therapy, particularly among those who fail to achieve successful conversion. These findings may support individualized surveillance and treatment strategies for RAS-mutant IU-CRLM.

## Introduction

Colorectal cancer (CRC) is the second leading cause of cancer-related mortality in the United States and ranks fifth in China (1, 2). More than half of CRC patients develop liver metastases (CRLM), for which surgical resection is considered the primary curative approach (3). However, approximately 80%–85% of patients with CRLM are deemed unresectable at initial diagnosis (4, 5). Currently, there is no consensus on the optimal systemic conversion therapy for unresectable CRLM (IU-CRLM). After systemic treatment, R0 resection rates in IU-CRLM range from 22% to 57 % (6). Although 20%–30% of resected patients can achieve a long-term overall survival benefit, most patients relapse within the first two years after hepatectomy, with about 35%–45% of early recurrences occurring in the first six months (7). Identifying clinical factors that strongly correlate with such early recurrence is therefore essential for guiding therapeutic decisions in this subgroup.

Previous research has shown that various clinicopathologic parameters—including advanced T stage, moderate histological differentiation, close or positive surgical margins in liver lesions, and elevated preoperative carcinoembryonic antigen (CEA) levels—are associated with poor prognosis in CRL M (8–11). While the metastatic capacity of CRC is primarily driven by oncogenic mutations, RAS mutations stand out as particularly common, with KRAS being the most frequent, occurring in approximately 40% of cases (12). KRAS-driven activation of the epidermal growth factor receptor (EGFR) pathway plays a crucial role in the malignant progression of CRL M (13, 14). The biological aggressiveness associated with RAS mutations may also be reflected in imaging heterogeneity. Recent radiomics-based studies have demonstrated that computed tomography features can non-invasively capture tumor heterogeneity and predict KRAS/NRAS/BRAF mutation status, suggesting that radiologic patterns may correlate with underlying molecular alterations in CRC (15). Numerous clinical studies have confirmed that KRAS mutation is a negative prognostic factor after CRLM resection (16–19). Our previous study also indicated that KRAS mutations predict an unfavorable prognosis in patients receiving bevacizumab-based conversion therapy (20). Nevertheless, whether KRAS mutations are associated with early progression in IU-CRLM remains unclear, as no relevant studies have been reported to date. Clarifying this relationship could aid in optimizing treatment strategies.

Against this background, the present study aims to characterize the RAS mutation spectrum in patients with IU-CRLM and to analyze the association between RAS mutation status, conversion outcomes, and early tumor progression. By doing so, we hope to generate additional evidence to guide clinical decision-making and improve therapeutic strategies for IU-CRLM.

## Methods

### Study population

This retrospective study consecutively included 194 patients diagnosed with CRLM through histological and radiological assessments, who received first-line treatment at Sun Yat-sen University Cancer Center between December 2012 and January 2020. The inclusion criteria were: (1) histologically confirmed colorectal adenocarcinoma with proficient mismatch repair (pMMR); (2) liver metastasis confirmed by radiological imaging, without evidence of other distant metastases; (3) unresectable CRLM at the time of liver metastasis diagnosis; (4) no prior history of liver resection or interventional treatment; (5) availability of RAS mutation status determined by Next-Generation Sequencing (NGS) or Polymerase Chain Reaction (PCR). Clinicopathological and follow-up data were collected by two independent research nurses through the hospital information system. RAS mutation testing was performed on DNA extracted from the primary tumor tissue of CRLM resections. All patients were screened for mutations in KRAS codons 12, 13, 61, and 146, as well as NRAS codons 12, 13, and 61, using conventional PCR-based primer extension methods. Mutations in any of these codons were collectively reported as RAS mutations. This study adhered to the Strengthening the Reporting of Observational Studies in Epidemiology (STROBE) Statement Guidelines (21).

Primary tumor differentiation was categorized as well/moderate differentiation vs. poor differentiation according to the pathological reports; poor differentiation in this study refers to poorly differentiated primary colorectal adenocarcinoma.

### Treatment strategy

The treatment strategy and resectability of liver metastases for each patient were determined by multidisciplinary team (MDT) consensus and individualized according to patient condition, economic considerations, and personal preference.

Targeted therapy was selected according to molecular testing results, primary tumor sidedness, contraindications, patient condition, drug availability, and MDT consensus. RAS status was mainly used to guide targeted therapy, particularly the use of anti-EGFR therapy, but did not solely determine the chemotherapy backbone.

Hepatectomy was performed only if the following criteria were met: (1) preservation of at least one of the three hepatic veins; (2) retention of more than 30%–40% of the residual liver volume; and (3) a resection margin of at least 1 mm. Tumor response or progression after first-line treatment was assessed according to the Response Evaluation Criteria in Solid Tumors (RECIST) (22). Radiofrequency ablation (RFA) or other ablative techniques were used for selected lesions with limited surgical accessibility, excessive operative morbidity, or small size, following MDT evaluation and consensus.

Successful conversion treatment was defined as liver metastases being deemed resectable or locally controllable following first-line systemic therapy, with patients achieving no evidence of disease (NED) status through curative-intent local treatment, including surgery alone, surgery combined with RFA/ablation, or RFA/ablation alone when applicable. Patients who underwent non-curative interventional procedures, ablation, or perfusion-related therapy without achieving NED were not classified as successfully converted. Failure of conversion treatment was defined as liver metastases remaining unresectable or not controllable by curative-intent local treatment after first-line therapy.

Early progression/recurrence was defined as the occurrence, within six months from the evaluation for conversion therapy, of any of the following events: (i) radiological or clinical disease progression after first-line treatment in patients whose conversion therapy failed, (ii) tumor recurrence in patients who achieved curative-intent local treatment and NED, or (iii) death from any cause. The early event endpoint was further separated into early progression after conversion failure, early recurrence after successful conversion, and early mortality within six months (23–25).

### Follow-up

For patients who underwent successful conversion treatment, follow-up visits were conducted every three months during the first two years after curative-intent local treatment and then every six months up to five years. Chest, abdominal, and pelvic computed tomography (CT) scans were performed at 3, 6, 12, 18, and 24 months, and annually thereafter. For patients with early progression/recurrence, follow-up visits were conducted every three months, with specific follow-up measures determined by the attending physician. All follow-ups were conducted by trained nurses, and carcinoembryonic antigen (CEA) levels were measured during each clinical evaluation. Liver magnetic resonance imaging (MRI) was performed to evaluate suspicious lesions identified on CT scans or in cases of negative CT results and elevated CEA levels.

Overall survival (OS) was calculated from the date of last systemic therapy to death from any cause or the last follow-up. Event-free survival (EFS) was defined as the interval from the evaluation for conversion therapy to the earliest of the following events: (i) radiological or clinical disease progression after first-line treatment in patients who experienced conversion failure, (ii) tumor recurrence in patients who achieved NED after curative-intent local treatment, or (iii) death from any cause. Patients without an event were censored at the date of their last follow-up. The last follow-up was conducted in January 2025.

### Statistical analysis

Statistical analyses were performed using Python 3.11 with the lifelines package for survival analysis, statsmodels for logistic regression, and matplotlib for figure generation. Categorical variables are presented as numbers (percentages), while continuous variables are expressed as medians with the first quartile (Q1) and third quartile (Q3). The chi-square test or Fisher's exact test was used to compare categorical variables, as appropriate. The Kaplan–Meier method and log-rank test were used to evaluate EFS and OS.

Multivariable logistic regression was applied to evaluate risk factors for early progression/recurrence. In addition to the original multivariable model based on variables with statistical significance in univariable analysis, an additional clinically adjusted model was constructed to further reduce confounding and increase model validity. This model included RAS status, primary tumor location, primary tumor differentiation, number of liver metastases, maximum size of liver metastases, bilobar involvement, baseline CEA level, first-line chemotherapy regimen, targeted therapy type, and conversion outcome. Subgroup logistic models were also performed for early progression after conversion failure and early recurrence after successful conversion when statistically feasible.

Cox proportional hazards models were used to assess factors associated with EFS and OS. A clinically adjusted Cox model was additionally constructed using the same clinically relevant covariates to evaluate the robustness of the association between RAS status and survival outcomes. Proportional hazards assumptions were assessed when applicable. All statistical tests were two-sided, and a *P*-value <0.05 was considered statistically significant.

## Results

### Clinical characteristics

Table 1 presents the clinical characteristics of 194 patients with IU-CRLM. Of these, 140 were male and 54 were female, and the median age was 55 years (Q1–Q3: 47–62 years). The primary tumor was located in the colon in 136 (70.1%) patients and in the rectum in 58 (29.9%) patients. A total of 98 (50.5%) patients had clinical T1–T3 disease, whereas 96 (49.5%) had clinical T4 disease. Among all participants, 166 (85.6%) exhibited lymph node metastasis, while 28 (14.4%) did not. RAS mutations were identified in 51 (26.3%) patients across the entire cohort.

**Table 1:** Patient demographics, tumour characteristics, and treatment in the total study population.

Variables	Total patients (*n* = 194)
Median age (range)—years	55.0 (47.0–62.0)
Gender—no. (%)	
Male	140 (72.2)
Female	54 (27.8)
Early progression/recurrence	
No	144 (74.2)
Yes	50 (25.8)
Primary Tumor characteristics	
Primary tumor location—no. (%)	
Left-side colon	94 (48.5)
Right-side colon	42 (21.6)
Rectum	58 (29.9)
Primary tumor differentiation—no. (%)	
Well or moderate	157 (80.9)
Poor	37 (19.1)
Liver metastasis	
Timing of metastasis—no. (%)	
Synchronous	181 (93.3)
Metachronous	13 (6.7)
Number of tumors—no. (%)	
≤8	100 (51.5)
>8	94 (48.5)
Maximum size of tumors—no. (%)	
≤6 cm	111 (57.2)
>6 cm	83 (42.8)
Distribution of tumors—no. (%)	
Unilobar	43 (22.2)
Bilobar	151 (77.8)
Staging	
Clinical T staging—no. (%)	
T1–T3	98 (50.5)
T4	96 (49.5)
Clinical N staging—no. (%)	
N0	28 (14.4)
N1–N2	166 (85.6)
Serum CEA (baseline)—no. (%)	
≤200 ng/mL	113 (58.2)
>200 ng/mL	81 (41.8)
Serum CA19-9 (baseline)—no. (%)	
≤200 U/mL	112 (57.7)
>200 U/mL	82 (42.3)
Total RAS status—no. (%)	
Wild type	143 (73.7)
Mutation	51 (26.3)
First-line chemotherapy regimens	
FOLFOX	129 (66.5)
FOLFIRI	29 (14.9)
FOLFOXIRI	36 (18.6)
Evaluation of efficacy	
PR	97 (50)
SD	52 (26.8)
PD	45 (23.2)
Targeted therapy—no. (%)	
Yes	153 (78.9)
No	41 (21.1)
Outcome of conversion therapy—no. (%)	
Success	87 (44.8)
Failure	107 (55.2)

CEA, carcinoembryonic antigen; CA19-9, carbohydrate antigen 19-9; PR, partial response; SD, stable disease; Progressive disease, PD; FOLFOX, oxaliplatin, 5-fluorouracil and leucovorin; FOLFIRI, lrinotecan, 5-fluorouracil and leucovorin; FOLFOXIRI, folinic acid, 5-fluorouracil, oxaliplatin and irinotecan.

Regarding first-line treatment, 129 (66.5%) patients were managed with FOLFOX, 29 (14.9%) with FOLFIRI, and 36 (18.6%) with FOLFOXIRI. Altogether, 153 (78.9%) patients received targeted therapy, of whom 99 (51.1%) underwent cetuximab-based treatment and 54 (27.8%) received bevacizumab. Based on genetic testing results, targeted therapy was selected accordingly: among the 99 patients who received cetuximab, 98 were RAS wild-type; among the 54 patients who received bevacizumab, 33 were RAS-mutant (Supplementary Table S3). Following first-line chemotherapy, 97 (50.0%) patients achieved a partial response (PR), 52 (26.8%) were classified as having stable disease (SD), and 45 (23.2%) demonstrated progressive disease (PD).

Ultimately, 87 (44.8%) patients underwent successful conversion therapy and achieved no evidence of disease (NED) through curative-intent local treatment. Among these patients, 42 underwent surgery alone, 34 underwent surgery combined with RFA/ablation, and 11 underwent RFA/ablation alone. Among the 107 patients with failed conversion, 86 received no curative local treatment, whereas 21 underwent non-curative intervention, ablation, or perfusion-related procedures and were not considered successfully converted (Supplementary Table S2).

During subsequent follow-up, 50 (25.8%) patients experienced early progression/recurrence, whereas 144 (74.2%) did not. By the final follow-up, 162 of the 194 patients (83.5%) experienced disease progression, and 113 (58.2%) died.

### Constitution of total RAS status and KRAS mutation

**Figure 1:**
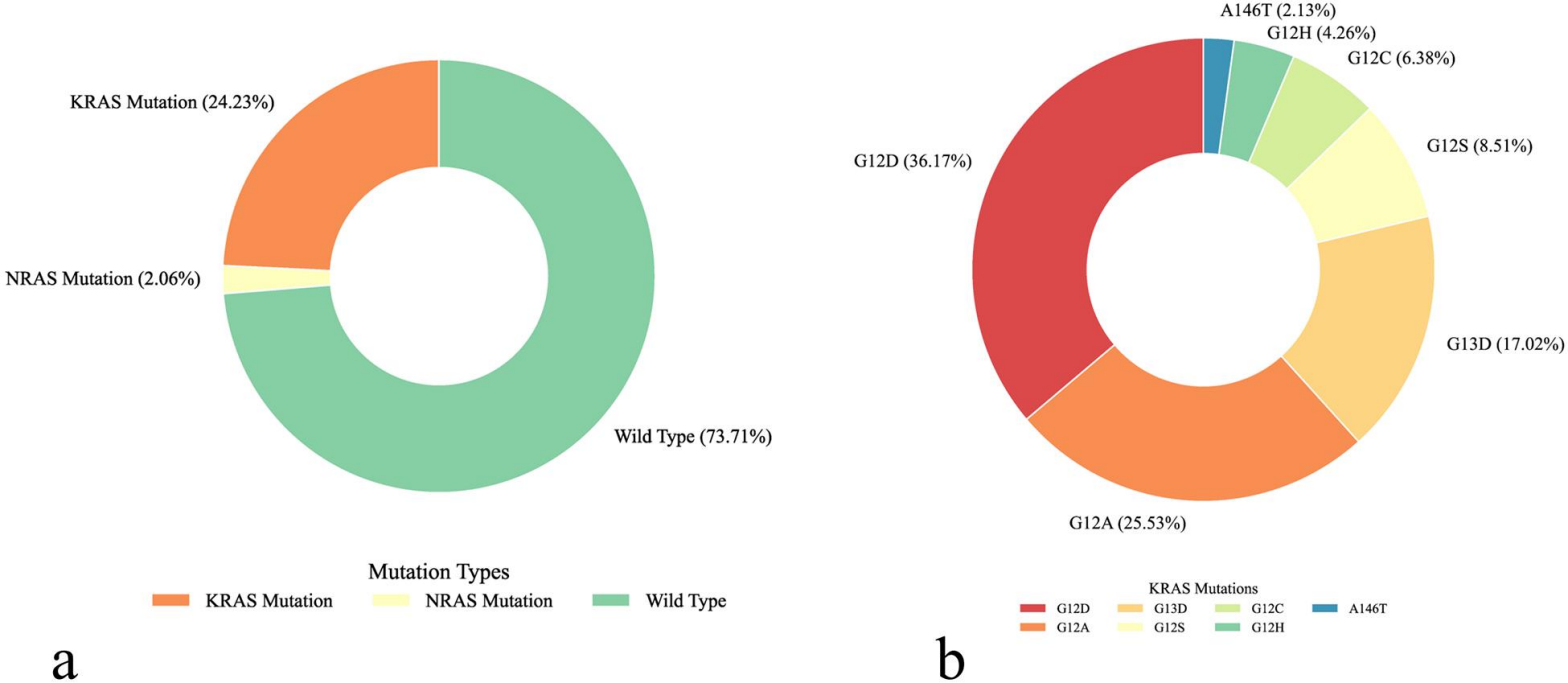
Overall distribution of RAS status and KRAS mutation subtypes. (**a**) Distribution of RAS status. (**b**) Distribution of KRAS-mutant subtypes.

An analysis of 194 prescreened cases indicated that total RAS status included KRAS mutation, NRAS mutation, and wild-type disease, with KRAS mutations accounting for 24.23% (Figure 1a). Roughly three quarters of the tumors were classified as wild-type. Further evaluation of KRAS mutations revealed seven distinct genotypes, with G12D, G12A, and G13D as the top three subtypes. The remaining subtypes collectively represented 21.28% (Figure 1b).

**Figure 2:**
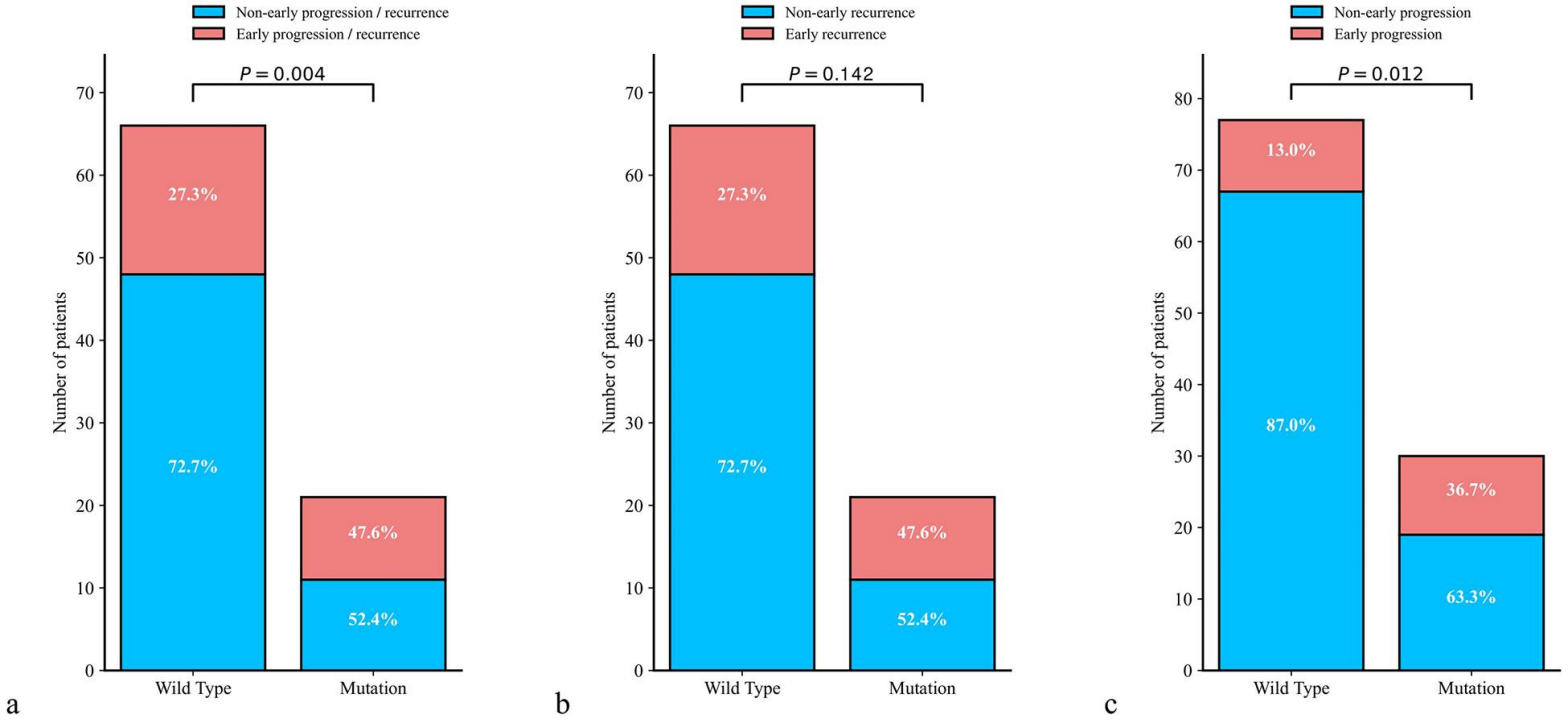
Relationship between RAS status and early disease progression/recurrence under different conversion therapy outcomes. (**a**) Comparison of early progression/recurrence rates between RAS-mutant and wild-type patients across the entire cohort. (**b**) Early recurrence rates in successfully converted patients, stratified by RAS status. (**c**) Early progression rates in the conversion-failure group, stratified by RAS status.

An additional assessment focused on RAS status in relation to early disease progression/recurrence according to different conversion therapy outcomes. Across the entire cohort, patients harboring RAS mutations had a higher incidence of early progression/recurrence than their wild-type counterparts (41.2% vs. 19.6%, *P* = 0.004, Figure 2a).

Early events were further separated into early progression after conversion failure, early recurrence after successful conversion, and early mortality within 6 months. Among patients with conversion failure, those harboring RAS mutations had a significantly higher early progression rate than RAS wild-type patients (36.7% vs. 13.0%, *P* = 0.012, Figure 2c). In contrast, among patients who achieved successful conversion, the difference in early recurrence rate between RAS-mutant and RAS wild-type patients did not reach statistical significance (47.6% vs. 27.3%, *P* = 0.142, Figure 2b). Early mortality within 6 months was uncommon and occurred in only 3 patients (3/194, 1.5%) (Supplementary Table S1).

Clinicopathological characteristics summarized in Table 2 revealed that RAS-mutant patients had a higher proportion of right-sided primary tumors than RAS wild-type patients (35.3% vs. 16.8%, *P* = 0.011). Regarding clinical staging, the fraction of patients with T4 disease (66.7% vs. 43.4%, *P* = 0.007) and N1–N2 disease (96.1% vs. 81.8%, *P* = 0.024) was notably larger in the RAS-mutant group. In addition, a higher percentage of RAS-mutant patients presented with baseline serum CA19-9 levels above 200 U/mL (56.9% vs. 37.1%, *P* = 0.022).

**Table 2:** Clinical characteristics stratified by total RAS status.

Variables	Wild type	Mutation	*P* Value
Age			0.959
≤60	95 (66.4)	33 (64.7)	0.117
>60	48 (33.6)	18 (35.3)	
Gender—no. (%)			
Male	108 (75.5)	32 (62.7)	
Female	35 (24.5)	19 (37.3)	
Primary tumor location—no. (%)			**0**.**011**
Left-side colon and Rectum	119 (83.2)	33 (64.7)	
Right-side colon	24 (16.8)	18 (35.3)	
Primary tumor differentiation—no. (%)			0.250
Well or moderate	119 (83.2)	38 (74.5)	
Poor	24 (16.8)	13 (25.5)	
Liver metastasis			
Timing of metastasis—no. (%)			0.550
Metachronous	11 (7.7)	2 (3.9)	
Synchronous	132 (92.3)	49 (96.1)	
Number of tumors—no. (%)			0.596
≤8	92 (64.3)	30 (58.8)	
>8	51 (35.7)	21 (41.2)	
Maximum size of tumors—no. (%)			0.154
≤6 cm	77 (53.8)	34 (66.7)	
>6 cm	66 (46.2)	17 (33.3)	
Distribution of tumors—no. (%)			0.752
Unilobar	33 (23.1)	10 (19.6)	
Bilobar	110 (76.9)	41 (80.4)	
Staging			
Clinical T staging—no. (%)			**0**.**007**
T1–T3	81 (56.6)	17 (33.3)	
T4	62 (43.4)	34 (66.7)	
Clinical N staging—no. (%)			**0**.**024**
N0	26 (18.2)	2 (3.9)	
N1–N2	117 (81.8)	49 (96.1)	
Serum CEA (baseline)—no. (%)			0.113
≤200 ng/mL	78 (54.5)	35 (68.6)	
>200 ng/mL	65 (45.5)	16 (31.4)	
Serum CA19-9 (baseline)—no. (%)			**0**.**022**
≤200 U/mL	90 (62.9)	22 (43.1)	
>200 U/mL	53 (37.1)	29 (56.9)	
Outcome of conversion therapy—no. (%)			0.653
Success	66 (46.2)	21 (41.2)	
Failure	77 (53.8)	30 (58.8)	

CEA, carcinoembryonic antigen; CA19-9, carbohydrate antigen 19-9.Bold values indicate statistically significant differences (*P* < 0.05) between RAS wild-type and RAS-mutant groups.

### Risk factors for early progression/recurrence in CRLMs

Table 3 summarizes the results of univariate and multivariate logistic regression analyses. In the univariate analysis, RAS mutant status (OR: 2.875, 95% CI: 1.436–5.755, *P* = 0.003), poor primary tumor differentiation (OR: 2.863, 95% CI: 1.345–6.093, *P* = 0.006), and failure of conversion therapy (OR: 1.944, 95% CI: 1.009–3.744, *P* = 0.047) were identified as significant risk factors for early progression/recurrence.

**Table 3:** Univariate and multivariate logistic regression analyses of risk factors for early progression/recurrence.

Parameters	Univariate		Multivariate	
	OR (95% CI)	*P* value	OR (95% CI)	*P* value
Age, years		0.106		
≤60	1.00			
>60	0.547 (0.263–1.138)			
Sex		0.385		
Male	1.00			
Female	0.732 (0.363–1.479)			
Primary tumor location		0.339		
Left-side colon and Rectum	1.00			
Right-side colon	0.692 (0.326–1.471)			
Primary tumor differentiation		**0**.**006**	3.066 (1.365–6.888)	**0**.**007**
Well or moderate	1.00			
Poor	2.863 (1.345–6.093)			
Timing of metastasis		0.851		
Synchronous	1.00			
Metachronous	1.136 (0.300–4.307)			
Number of metastatic tumors		0.456		
≤8	1.00			
>8	0.781 (0.408–1.495)			
Distribution of liver metastasis		0.651		
Unilobar	1.00			
Bilobar	0.838 (0.391–1.798)			
Median size of largest liver tumor—cm		0.748		
≤6	1.00			
>6	0.898 (0.465–1.732)			
Clinical T staging		0.364		
T1–T3	1.00			
T4	1.352 (0.705–2.591)			
Clinical N staging		0.615		
N0	1.00			
N1–N2	1.282 (0.487–3.372)			
Serum CEA		0.137		
≤200 ng/mL	1.00			
>200 ng/mL	0.597 (0.302–1.179)			
Serum CA19-9		0.923		
≤200 U/mL	1.00			
>200 U/mL	1.033 (0.537–1.988)			
Total RAS status		**0**.**003**	2.906 (1.401–6.027)	**0**.**004**
Wild type	1.00			
Mutation	2.875 (1.436–5.755)			
First-line chemotherapy regimens		0.580		
FOLFOX/FOLFIRI	1.00			
FOLFOXIRI	1.211 (0.615–2.383)			
Targeted therapy		0.794		
Yes	1.00			
No	0.901 (0.412–1.970)			
Outcome of conversion therapy		**0**.**047**	2.385 (1.178–4.826)	**0**.**016**
Success	1.00			
Failure	1.944 (1.009–3.744)			

CEA, carcinoembryonic antigen; CA19-9, carbohydrate antigen 19-9; FOLFOX, oxaliplatin, 5-fluorouracil and leucovorin; FOLFIRI, lrinotecan, 5-fluorouracil and leucovorin; FOLFOXIRI, folinic acid, 5-fluorouracil, oxaliplatin and irinotecan.Bold values indicate statistically significant associations (*P* < 0.05). OR, odds ratio; CI, confidence interval.

The multivariate analysis further demonstrated that RAS mutant status (OR: 2.906, 95% CI: 1.401–6.027, *P* = 0.004) and poor primary tumor differentiation (OR: 3.066, 95% CI: 1.365–6.888, *P* = 0.007) were significantly associated with an increased risk of early progression/recurrence. Failure of conversion therapy was also associated with an increased risk of early progression/recurrence (OR: 2.385, 95% CI: 1.178–4.826, *P* = 0.016).

To further reduce confounding and improve model validity, an additional clinically adjusted model was constructed by including the number of liver metastases, maximum diameter of liver metastases, bilobar involvement, baseline CEA level, primary tumor location, chemotherapy regimen, targeted therapy type, and conversion outcome. In this model, RAS mutation remained independently associated with early progression/recurrence in the overall cohort (OR: 3.546, 95% CI: 1.290–9.752, *P* = 0.014). In the conversion-failure subgroup, RAS mutation also remained significantly associated with early progression after adjustment (OR: 10.218, 95% CI: 1.904–54.825, *P* = 0.007), whereas no significant association was observed between RAS mutation and early recurrence after successful conversion (OR: 1.484, 95% CI: 0.327–6.728, *P* = 0.609) (Supplementary Table S4).

### Survival analysis in patients with initially unresectable colorectal cancer liver metastases

**Figure 3:**
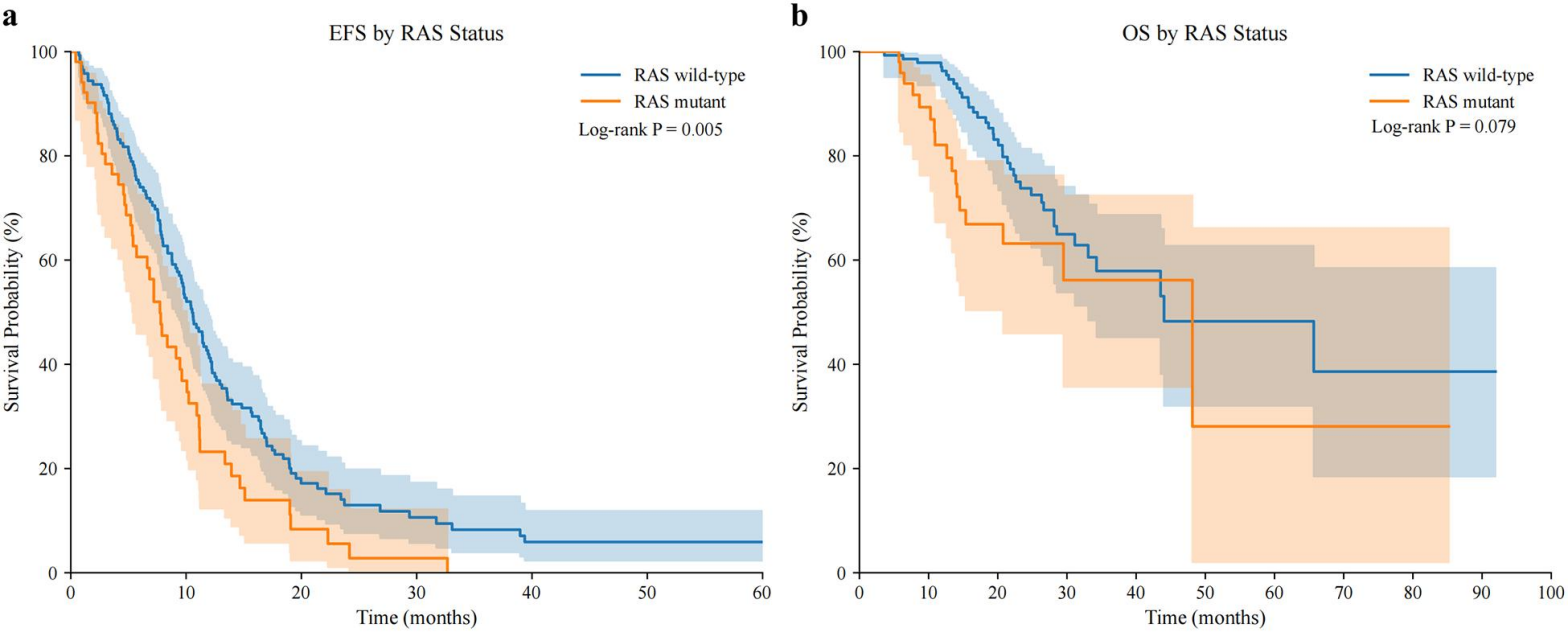
Comparison of event-free survival (EFS) and overall survival (OS) between RAS-mutant and wild-type CRLM patients. (**a**) EFS by RAS status. (**b**) OS by RAS status.

The median follow-up period was 30.5 months. Kaplan–Meier analysis showed that patients with RAS-mutant tumors had poorer EFS than those with RAS wild-type tumors (log-rank *P* = 0.005, Figure 3a). OS analysis is shown in Figure 3b (log-rank *P* = 0.079).

**Figure 4:**
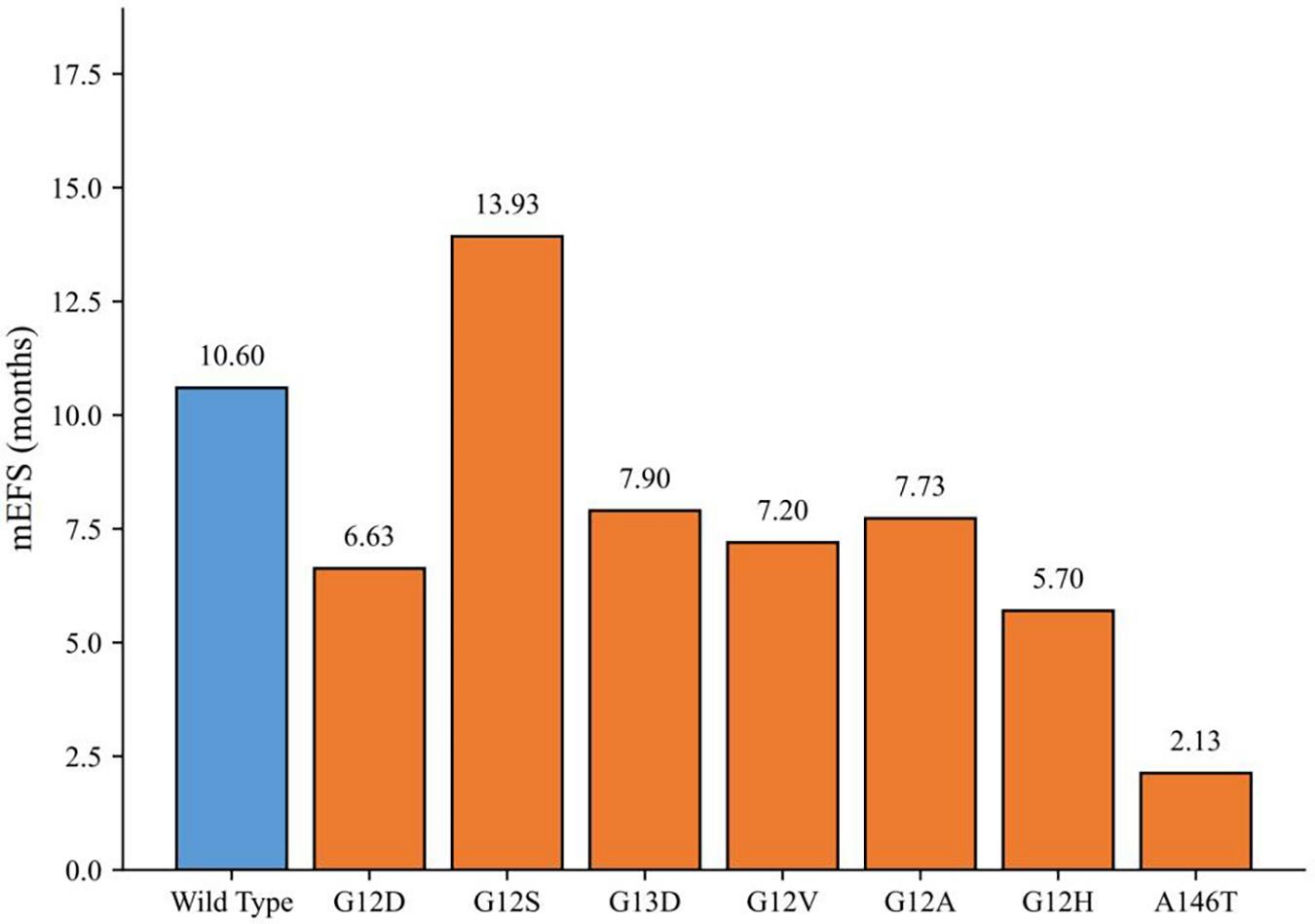
Exploratory subgroup analysis of median event-free survival (mEFS) according to KRAS mutation subtype.

An exploratory analysis of KRAS mutation subtypes showed that patients with KRAS wild-type tumors had a median EFS of 10.60 months. Because of the small sample size of several KRAS mutation subtypes, subtype-specific findings were considered exploratory (Figure 4).

In Cox regression analysis, primary tumor differentiation (*P* = 0.040), primary tumor location (*P* = 0.004), presentation of liver metastases (*P* = 0.027), number of tumors >8 (*P* = 0.021), and RAS mutation (*P* = 0.007) were significantly associated with adverse EFS (Table 4). In the multivariable model, RAS mutation (HR: 1.518, 95% CI: 1.053–2.187, *P* = 0.025) and poor primary tumor differentiation (HR: 1.591, 95% CI: 1.076–2.352, *P* = 0.020) remained independently associated with poorer EFS. To further reduce confounding and improve model validity, additional clinically adjusted Cox models were constructed and are presented in Supplementary Table S4. In these supplemental models, the association between RAS mutation and EFS remained directionally consistent but was attenuated after adjustment (HR: 1.476, 95% CI: 0.933–2.335, *P* = 0.096), while RAS mutation was associated with worse OS (HR: 2.385, 95% CI: 1.094–5.199, *P* = 0.029).

**Table 4:** Univariate and multivariate cox regression analyses of risk factors for event-free survival.

Characteristics	Univariable		Multivariable	
	HR (95% CI)	*P* value	HR (95% CI)	*P* value
Age (>60 years vs. ≤60 years)	0.820 (0.589–1.144)	0.243		
Sex (male vs. female)	0.804 (0.568–1.138)	0.219		
Primary tumour location (left colon vs. right colon and rectum)	0.581 (0.401–0.841)	0.004	0.690 (0.470–1.014)	0.059
Primary tumour differentiation (poor vs. well to moderate)	1.496 (1.018–2.198)	0.040	1.591 (1.076–2.352)	0.020
Presentation of liver metastases (synchronous vs. metachronous)	2.242 (1.094–4.592)	0.027	2.060 (0.992–4.278)	0.053
Distribution of tumors (baseline)	1.111 (0.762–1.619)	0.585		
Number of tumors (>8 vs. ≤8)	1.446 (1.058–1.978)	0.021	1.364 (0.995–1.871)	0.054
Maximum size of tumors (>6 cm vs. ≤6 cm)	1.186 (0.867–1.623)	0.287		
Clinical T staging (T4 vs. T1–3)	1.245 (0.911–1.702)	0.169		
Clinical N staging (N1–2 vs. N0)	1.248 (0.807–1.929)	0.320		
Total RAS (mutation vs. wild-type)	1.629 (1.144–2.321)	0.007	1.518 (1.053–2.187)	0.025
Targeted therapy (yes vs. no)	0.898 (0.612–1.318)	0.582		
Conversion outcome (failure vs. success)	0.986 (0.722–1.347)	0.931		
First-line chemotherapy regimens (FOLFOX or FOLFIRI vs. FOLFOXIRI)	1.106 (0.973–1.258)	0.123		
Serum CEA (≤200 ng/mL vs. >200 ng/mL)	0.973 (0.711–1.332)	0.865		
Serum CA19-9 (≤200 U/mL vs. >200 U/mL)	1.236 (0.902–1.694)	0.187		

CEA, carcinoembryonic antigen; CA19-9, carbohydrate antigen 19-9; FOLFOX, oxaliplatin, 5-fluorouracil and leucovorin; FOLFIRI, lrinotecan, 5-fluorouracil and leucovorin; FOLFOXIRI, folinic acid, 5-fluorouracil, oxaliplatin and irinotecan.

## Discussion

KRAS and NRAS alterations are well-recognized molecular markers in metastatic colorectal cancer and are routinely used to guide the selection of targeted therapy, particularly anti-EGFR therapy (26, 27). In the present cohort, RAS mutations were detected in 26.3% of patients with initially unresectable CRLM, with KRAS accounting for most RAS mutations. Consistent with previous studies reporting the adverse prognostic impact of RAS/KRAS mutations in CRLM, RAS-mutant tumors in the present cohort were associated with several adverse clinicopathologic features, including a higher proportion of right-sided primary tumors, more advanced clinical T and N stages, and a higher proportion of elevated baseline CA19-9 levels (13, 16–20).

The principal finding of this study is that RAS mutation was associated with an increased risk of early progression/recurrence in patients with IU-CRLM undergoing conversion therapy. In the overall cohort, RAS-mutant patients showed a higher early progression/recurrence rate than RAS wild-type patients. After additional clinical adjustment for tumor burden, bilobar involvement, CEA level, primary tumor location, chemotherapy regimen, targeted therapy, and conversion outcome, RAS mutation remained independently associated with early progression/recurrence. These findings support the value of incorporating RAS status into risk stratification for early disease failure in this high-risk population.

Early recurrence within six months has been recognized as a clinically meaningful adverse event after CRLM treatment (7, 8, 23–25). Because early progression/recurrence in this study included biologically distinct events, we further separated early events into early progression after failed conversion, early recurrence after successful conversion, and early mortality within six months. RAS mutation was significantly associated with early progression in patients with failed conversion, whereas the difference in early recurrence between RAS-mutant and RAS wild-type patients did not reach statistical significance among successfully converted patients. Early mortality within six months was uncommon. These results suggest that the adverse effect of RAS mutation may be concentrated mainly in patients who fail to achieve successful conversion.

The subgroup findings also highlight the potential clinical importance of achieving NED through curative-intent local treatment, consistent with multidisciplinary concepts of resectability and ablatability in liver-only CRLM (3, 4, 9). Among successfully converted patients, local treatment included surgery alone, surgery combined with RFA/ablation, and RFA/ablation alone; patients who underwent non-curative intervention, ablation, or perfusion-related procedures without achieving NED were not classified as successfully converted. Although these results should not be interpreted as evidence that local therapy eliminates the biological effect of RAS mutation, they suggest that successful conversion may attenuate the short-term adverse prognostic impact of RAS mutation.

Exploratory analyses of KRAS mutation subtypes suggested heterogeneity in EFS among different subtypes, which is biologically plausible given known functional differences and prognostic heterogeneity among KRAS variants (12, 28). Previous studies have suggested that different KRAS mutation sites and subtypes may generate distinct clinical phenotypes by affecting GTP hydrolysis, nucleotide exchange, downstream signaling intensity, and tumor biological behavior (12, 28). However, the number of patients in several KRAS subtype groups was small, and the subtype-specific findings should therefore be interpreted cautiously. In the current revision, KRAS subtype results are retained as descriptive exploratory findings rather than as a basis for definitive prognostic conclusions.

From a treatment-strategy perspective, our findings suggest that patients with RAS-mutant tumors who fail to achieve conversion may require more proactive subsequent therapy and closer surveillance. Previous randomized trials have shown that, for selected patients with metastatic colorectal cancer who are suitable for intensive treatment, FOLFOXIRI plus bevacizumab can deepen tumor response and improve treatment outcomes, supporting its clinical value in patients with high tumor burden or a clear need for tumor shrinkage and conversion (29, 30). In addition, with the emerging antitumor activity of KRAS G12C inhibitors combined with anti-EGFR therapy in KRAS G12C-mutant metastatic colorectal cancer, subsequent precision treatment options for patients with RAS/KRAS-mutant disease are expanding (31). However, whether these strategies can reduce the risk of early progression in patients with IU-CRLM remains to be validated in prospective studies.

This study has several limitations. First, it is a single-center retrospective analysis and is therefore subject to inherent selection bias. Treatment selection was influenced by MDT decision-making, patient condition, economic considerations, drug availability, and patient preference; therefore, treatment-selection bias could not be fully avoided. Second, although additional analyses were performed to separate early progression, early recurrence, and early mortality, the number of early deaths was small, and some subgroup analyses had limited statistical power. Third, local treatment modalities differed among successfully converted patients, and the RFA/ablation-alone subgroup was relatively small. Finally, although all patients underwent RAS genotyping, the small sample sizes of several KRAS subtypes limit the robustness of subtype-specific conclusions.

Overall, RAS mutation was associated with a higher risk of early progression/recurrence in patients with IU-CRLM receiving conversion therapy, particularly among those who failed to achieve successful conversion. The association between RAS mutation and EFS remained directionally consistent after additional clinical adjustment, although statistical significance was attenuated, while RAS mutation was associated with worse OS in the clinically adjusted model. These findings support more cautious surveillance and individualized subsequent treatment strategies for RAS-mutant patients, especially in the setting of failed conversion.

## Conclusions

In conclusion, RAS mutation was identified as an independent risk factor for early progression/recurrence in patients with IU-CRLM receiving systemic therapy. Notably, in patients with failed conversion therapy, the presence of RAS mutations was associated with a higher likelihood of early progression, underscoring the importance of a more cautious and individualized treatment and surveillance strategy by the MDT team for this high-risk subgroup.

## Data Availability

The authenticity of this article has been confirmed by uploading the key raw data to the Research Data Deposit public platform (www.researchdata.org.cn). These data are available upon request from the corresponding author.
