# Supplementary material for: Impact of RAS mutation status on early progression in patients with initially unresectable colorectal liver metastases undergoing conversion therapy: a retrospective analysis
Source: Front Surg. 2026 Jul 1;13:1853947. doi: 10.3389/fsurg.2026.1853947 (PMC13369114; doi:10.3389/fsurg.2026.1853947)
Supplement: Supplementary file 1 [file Datasheet1.pdf]

Supplementary Tables

Supplementary Table S1. Early event analyses stratified by conversion outcome.

Population	Endpoint	Overall	RAS wild-type	RAS mutant	P value
All patients	Early progression/recurrence	49/194 (25.3%)	28/143 (19.6%)	21/51 (41.2%)	0.004
Conversion failure	Early progression	21/107 (19.6%)	10/77 (13.0%)	11/30 (36.7%)	0.012
Successful conversion	Early recurrence	28/87 (32.2%)	18/66 (27.3%)	10/21 (47.6%)	0.142
All patients	Early mortality within 6 months	3/194 (1.5%)	1/143 (0.7%)	2/51 (3.9%)	—

Abbreviations: RAS, rat sarcoma viral oncogene homolog; CI, confidence interval. P values for RAS comparisons are shown where applicable. Percentages in the first three rows follow the manuscript/Figure 2 reporting convention.

Supplementary Table S2. Local treatment patterns according to conversion outcome.

Conversion outcome	Local treatment category	N	RAS wild-type	RAS mutant	Early event, n (%)	Early mortality within 6 months
Successful conversion	Surgery alone	42	33	9	12 (28.6%)	0
Successful conversion	Surgery + RFA/ablation	34	24	10	16 (47.1%)	0
Successful conversion	RFA/ablation alone	11	9	2	0 (0.0%)	0
Conversion failure	No curative local treatment	86	61	25	18 (20.9%)	3
Conversion failure	Non-curative intervention / ablation / perfusion-related procedure	21	16	5	3 (14.3%)	0

Abbreviations: RFA, radiofrequency ablation; NED, no evidence of disease. One successfully converted patient with incomplete local-treatment classification was confirmed as having undergone surgery and was incorporated into the surgery-alone group.

Supplementary Table S3. Treatment selection and treatment-related variables according to RAS status.

Variable	Category	RAS wild-type, n (%)	RAS mutant, n (%)	P value
First-line chemotherapy regimen	FOLFOX/XELOX/FOLFOX+FUDR	92 (64.3%)	35 (68.6%)	0.208
	FOLFIRI/other	18 (12.6%)	2 (3.9%)	
	FOLFOXIRI/CPT-11-OX-FUDR	33 (23.1%)	14 (27.5%)	
Targeted therapy	No targeted therapy	24 (16.8%)	17 (33.3%)	<0.001
	Cetuximab	98 (68.5%)	1 (2.0%)	
	Bevacizumab	21 (14.7%)	33 (64.7%)	
Conversion outcome	Conversion failure	77 (53.8%)	30 (58.8%)	0.653
	Successful conversion	66 (46.2%)	21 (41.2%)	
Response evaluation	PR	74 (51.7%)	23 (45.1%)	0.717
	SD	37 (25.9%)	15 (29.4%)	
	PD	32 (22.4%)	13 (25.5%)	
Primary tumor site	Left-sided colon and rectum	119 (83.2%)	33 (64.7%)	0.011
	Right-sided colon	24 (16.8%)	18 (35.3%)	

Abbreviations: PR, partial response; SD, stable disease; PD, progressive disease; FOLFOX, oxaliplatin, 5-fluorouracil and leucovorin; FOLFIRI, irinotecan, 5-fluorouracil and leucovorin; FOLFOXIRI, folinic acid, 5-fluorouracil, oxaliplatin and irinotecan. P values were calculated using chi-square tests.

Supplementary Table S4. Additional multivariable models with clinical adjustment.

Analysis type	Population / endpoint	Model	Effect measure for RAS mutation	Estimate	95% CI	P value
Logistic regression	Full cohort / early progression or recurrence	Core model	OR	3.208	1.547–6.650	0.002
Logistic regression	Full cohort / early progression or recurrence	Clinically adjusted model	OR	3.546	1.290–9.752	0.014
Logistic regression	Full cohort / early progression or recurrence	Extended model	OR	4.316	1.420–13.121	0.010
Logistic regression	Conversion failure / early progression	Core model	OR	4.219	1.482–12.011	0.007
Logistic regression	Conversion failure / early progression	Clinically adjusted model	OR	10.218	1.904–54.825	0.007
Logistic regression	Conversion failure / early progression	Extended model	OR	14.240	2.088–97.121	0.007
Logistic regression	Successful conversion / early recurrence	Core model	OR	2.588	0.915–7.320	0.073
Logistic regression	Successful conversion / early recurrence	Clinically adjusted model	OR	1.484	0.327–6.728	0.609
Logistic regression	Successful conversion / early recurrence	Extended model	OR	2.175	0.404–11.714	0.366
Cox regression	EFS	Selected multivariable model	HR	1.439	1.000–2.071	0.050
Cox regression	EFS	Clinically adjusted model	HR	1.476	0.933–2.335	0.096
Cox regression	OS	Clinically adjusted model	HR	2.385	1.094–5.199	0.029

Abbreviations: OR, odds ratio; HR, hazard ratio; CI, confidence interval; EFS, event-free survival; OS, overall survival. Core logistic models included RAS status, primary tumor differentiation, and conversion outcome when applicable. Clinically adjusted logistic and Cox models included RAS status, primary tumor site, primary tumor differentiation, number of liver metastases, maximum tumor size, bilobar involvement, baseline CEA, chemotherapy regimen, targeted therapy, and conversion outcome when applicable. Extended logistic models additionally included age, sex, clinical T stage, clinical N stage, and CA19-9.
